# Underexpression of Deleted in liver cancer 2 (DLC2) is associated with overexpression of RhoA and poor prognosis in hepatocellular carcinoma

**DOI:** 10.1186/1471-2407-8-205

**Published:** 2008-07-23

**Authors:** Li Xiaorong, Wu Wei, Qian Liyuan, Yang Kaiyan

**Affiliations:** 1 Department of General Surgery, The Third Affiliated Hospital of Central South University, Central South University, Tongzhipo Road, Changsha, Hunan Province, 410013, PR China

## Abstract

**Background:**

DLC2, a unique RhoGAP, has been recently identified as a tumor suppressor gene in hepatocellular carcinoma (HCC). However, the expression of DLC2 protein, and its relationship with RhoA in clinical hepatocellular carcinoma have not been studied. The aim of this study was to investigate the DLC2 protein expression and its correlation with expression of RhoA, as well as to evaluate the prognostic value of DLC2 for HCC patients.

**Methods:**

Western blot and immunohistochemical staining were employed to detect DLC2 protein expression in 128 HCC specimens. The correlation between DLC2 protein expression and clinicopathologic outcome, and prognostic value of DLC2 for HCC patients were analyzed.

**Results:**

HCC tissues revealed significantly lower level of DLC2 protein than pericarcinomatous liver tissues (PCLT). There was significant correlation between underexpression of DLC2 protein and cell differentiation. Meanwhile, underexpression of DLC2 protein was correlated with overexression of RhoA. Furthermore, HCC Patients with DLC2-negative expression showed a significantly poorer prognosis than those with DLC2-positve expression.

**Conclusion:**

Our data strongly suggested that decreased DLC2 expression in HCC correlates with cell differentiation of HCC and overexpression of RhoA, underexpression of DLC2 is associated with poor prognosis in HCC patients.

## Background

Hepatocellular carcinoma (HCC) is one of the most common malignancies in Asia and Africa, especially in China [1,2]. It is responsible for approximately one million deaths each year, predominantly in the developing countries [3]. During the past decades, hepatic resection for HCC has evolved into a safe procedure with low operative mortality [4,5]. However, the molecular mechanisms leading to the development and progression of hepatocellular carcinoma remains unclear. Thus, the delineation of the mechanisms for hepatocarcinogenesis is of importance, because it provides novel opportunities for diagnosis, prognosis, and therapeutic interventions.

RhoA-GTPase is a member of the Ras-superfamily of small guanosine triphosphatases (GTPases), which shuttles between an inactive GDP-bound state and an active GTP-bound state and exhibits intrinsic GTPase activities [6]. Activation of Rho protein causes to assembly of the actin-myosin contractile filaments into focal adhesion complexes that leads to cell polarity and facilitate motility [7]. In human cancers, the alteration of RhoA expression is involved in tumorigenesis. Indeed, overexpression of RhoA was detected in several types of cancer including bladder, testicular, ovarian, colon, breast, and lung [8-11]. Our previous study revealed that overexpression of RhoA was associated with poor prognosis in hepatocellular carcinoma [12]. As activating of RhoGTPases can stimulate cell proliferation and cell motility, inhibition of the RhoA activity may suppress the oncogenic and metastatic potential of tumor cells. Recently, a unique RhoGAP, which named DLC2 (deleted in liver cancer 2) because of its high homology to tumor suppressor gene DLC1, has been identified [13]. DLC2 contains a RhoGAP domain and exhibits GAP activity on RhoA and Cdc42 in vitro. Researches show that DLC2 suppresses cell transformation by means of inhibition of RhoA activity in hepatocellular carcinoma cells [14], meanwhile DLC2 mRNA is underexpressed in human hepatocellular carcinoma, which suggested a potential prognostic value of DLC2 for HCC patients.

However, there has been no available data on the protein expression of DLC2 in clinical hepatocellular carcinoma. Does DLC2 protein underexpress in hepatocellular carcinoma? Or does its expression correlate with clinicopathological parameters of HCC and expression of RhoA? Especially, whether DLC2 is a valuable prognostic marker for HCC patients? In this study, therefore, we examined the DLC2 protein expression in HCC and evaluated the relationship between DLC2 expression and clinicopathological parameters of HCC. Meanwhile, we investigated the relationship between expression of DLC2 and RhoA; most importantly, the prognostic value of DLC2 for HCC patients was also investigated.

## Methods

### Tissue specimens

The study protocol was approved by the Ethics Committee of the Central South University. Fresh samples of HCC tissue and pericarcinomatous liver tissue (PCLT, 1 cm away from the carcinoma) were obtained from 53 (46 male and 7 female) patients with primary hepatocellular carcinoma who underwent curative hepatectomy at the Third Affiliated Hospital of Central South University (CSU) during 2000 to 2003. The specimens were immediately frozen in liquid nitrogen and stored at -80°C for western blotting. The median age of these patients was 55 year, ranging from 19~75 year. All specimens obtained from hepatic resection were confirmed by pathological examination and clinicopathological parameters such as tumor diameter, number of tumor node, tumor capsule, histopathological classification, venous invasion, extrahepatic metastatic lesion and hepatitis virus infection were obtained.

### Antibody

Because there is no commercial DLC2 antibody at present, a mouse anti-human DLC2 monoclonal antibody was developed through ProMab Company. Brief, a 15aa polypeptide representing a unique epitope was synthesized according to the full-length DLC2 sequence [15], then the polypeptide was coupled with KLH. After injection in mouse, immune serum was obtained following standard techniques. Western blot confirmed the specificity of the antibody for DLC2. The quality control examination showed that the antibody is valid.

### Western blotting

Tissues from HCC and PCLT were lysed in a lysis buffer (Sigma, St. Louis, MO, USA) and protease-inhibitor (Promega, Madison, USA). The lysates were centrifuged at 13,000 g for 20 min at 4°C and the supernatants were stored at -80°C. Extracts equivalent to 50 μg of total protein were separated by SDS-PAGE gel and were blotted to polyvinylidene fluoride membrane (Sigma, St. Louis, MO, USA). After they were blocked in 4% nonfat dry milk in PBS containing 0.1% Tween-20 for 1 h at room temperature, the membranes were incubated with primary antibody (mouse anti-human DLC2 monoclonal antibody, ProMab, China, diluted at 1:500) for 1 h at 37°C. After washing, the membranes were incubated with a 1:5000 dilution of horseradish peroxidase-linked rabbit anti-mouse antibody (ProMab, China) for 30 mins at 37°C. Then the membranes were washed and treated with western blotting luminal reagent (Santa Cruz, California, USA) to visualize the bands, the results were obtained on Kodak film and quantified by densitometry (Beckman, South Pasadena, Canada) [16].

### Immunohistochemistry

One hundred and twenty-eight HCC deparaffinized specimens, including the 53 cases of HCC fresh specimens used for Western blotting, were evaluated for immunohistochemistry. All the specimens were collected from the Third Affiliated Hospital of Central South University (CSU) between 1991 and 2003. The clinicopathological parameters of all the specimens, including tumor diameter, number of tumor node, tumor capsule, histopathological classification, venous invasion, extrahepatic metastatic lesion and hepatitis virus infection were obtained. Follow-up data was obtained following the operations for all patients; the endpoint of the follow-up was set at the patients' death. In brief, tissue sections of 4 μm thick were cut and baked at 60°C for 2 h, deparaffinized in xylene and rehydrated through graded ethanol. Next, 3% hydrogen peroxide was applied to block the endogenous peroxidases for 20 mins, and the sections were subjected to heat-induced antigen retrieval in 0.01 M citrate buffer (PH = 6.0). The sections were incubated with normal rabbit serum to reduce non-specific binding. Then, they were incubated at 37°C for 1 h with specific antibodies (mouse anti-human DLC2 monoclonal antibody, ProMab, China) used at a 1:50 dilution. The second antibody was applied for 45 min at 37°C. The Streptavidin-biotin-peroxidase complex (SABC) tertiary system (Boster, Wuhan, China) was used according to the manufacturer's instruction for 20 min at room temperature. The tissues were visualized by applying 3, 3-diaminobenzidine tetrahydrochloride (DAB) for 3 min. Sections were counterstained using haematoxylin, dehydrated through gradient alcohols and mounted for viewing. Negative controls were done by omitting the primary antibody, whereas, HCC specimens of high DLC2 expression confirmed by Western blotting were used as positive controls. The intensity of cytoplasmic staining was scored as 0 to 3+ by comparison to the positive controls. DLC2-negative expressions were devoid of any cytoplasmic staining or contained faint, equivocal staining (scores 0 and 1+) [12].

### Statistical analysis

Statistical analysis was performed using the SPSS (version 11.0, Chicago, IL). A Student's t test was used to analyze the expression of DLC2 between HCC and PCLT. Spearman's correlation coefficient was used to examine the relationship between expression of DLC2 and RhoA. The Mann-Whitney U test and chi-square test were monitored to analyze the correlation between the expression of DLC2 and clinicopathological variables. Post-operative overall survival was analyzed by the Log-rank test. Differences were considered significant when *P *< 0.05.

## Results

### Expression of DLC2 protein in HCC and PCLT

**Figure 1:**
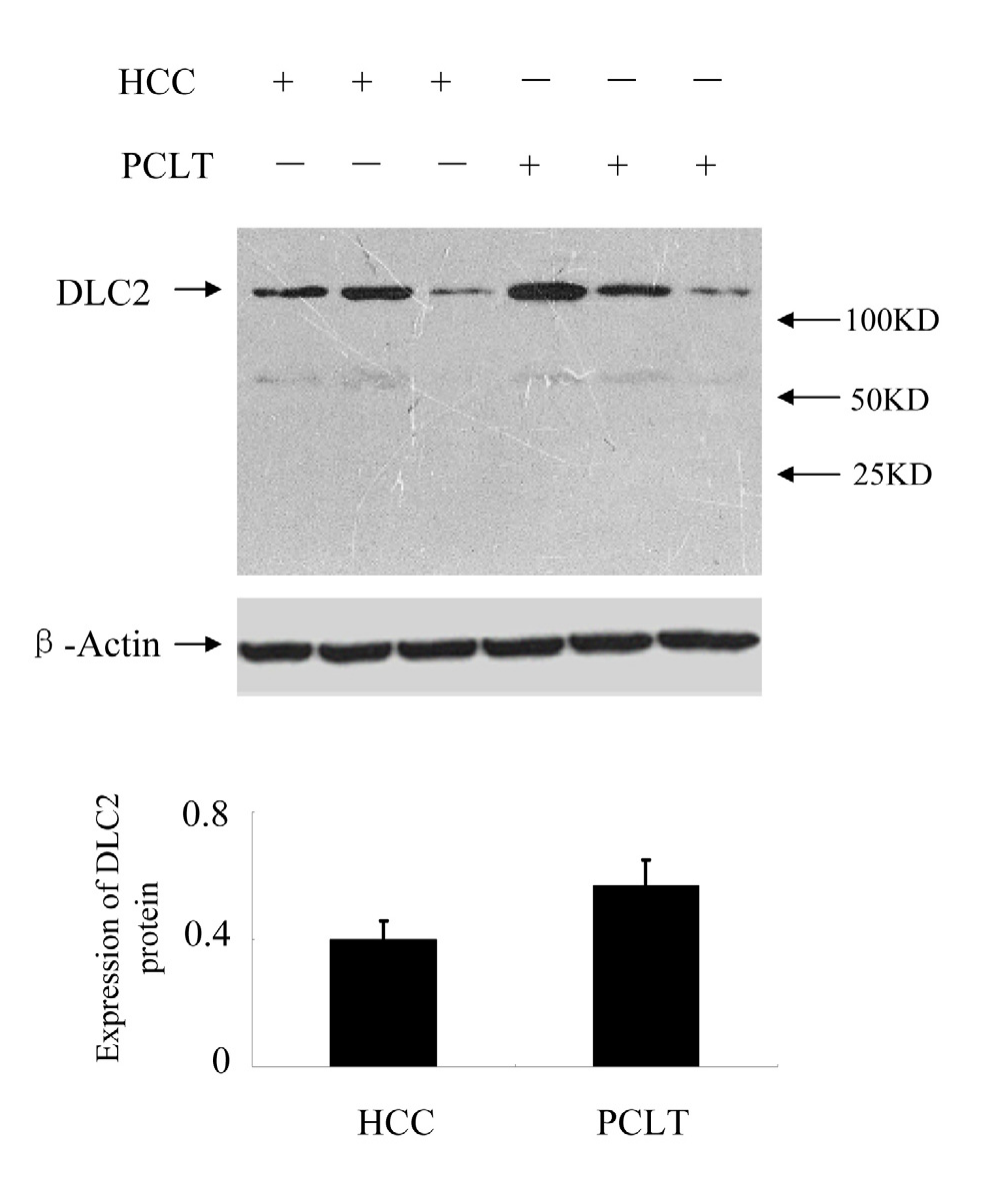
**Expression of DLC2 protein**. Expression of DLC2 protein was detected in 53 cases of HCC fresh specimens using western blot, HCC tissues revealed significantly lower levels of DLC2 protein than PCLT. *P *= 0.02.

Expression of DLC2 protein was detected in all HCC and PCLT. The relative expression of DLC2 protein was 0.40 ± 0.06 in HCC and 0.57 ± 0.08 in PCLT respectively. HCC tissues revealed significantly lower levels of DLC2 protein than PCLT (*P *= 0.02) (Figure 1).

### Correlation between DLC2 expression levels and clinicopathological parameters of HCC

The distribution pattern of DLC2 was determined by dividing the expression levels of DLC2 protein in subgroups by clinicopathological parameters. HCC with poor cell differentiation showed a lower expression level of DLC2 protein than those with well cell differentiation. There were no significant associations between expression of DLC2 gene and other clinicopathological parameters such as sex, hepatitis virus infection, liver cirrhosis, tumor size and capsular condition, venous invasion and nodular number (Table 1). The same result was observed based on the correlation between immunohistochemical study and clinicopathological parameters of 128 cases of HCC specimen (Table 2).

**Table 1:** The relationship between expression level of DLC2 protein and clinicopathological parameters for 53 cases of HCC specimen.

Clinicopathological parameters	No. of patients	Expression of DLC2 protein	*P *value
Sex			
Male	46	0.41 ± 0.08	0.49
Female	7	0.39 ± 0.06	
Liver cirrhosis			
Present	32	0.39 ± 0.04	0.78
Absent	21	0.40 ± 0.07	
Hepatitis virus infection			
Present	47	0.41 ± 0.04	0.72
Absent	6	0.40 ± 0.05	
Capsule formation			
Present	27	0.40 ± 0.04	0.63
Absent	26	0.39 ± 0.06	
Tumor nodule			
≥ 2	35	0.42 ± 0.10	0.23
< 2	18	0.39 ± 0.06	
Cell differentiation*			
I ~ II	24	0.42 ± 0.06	0.02**
III ~ IV	29	0.38 ± 0.05	
Vein invasion			
Present	26	0.41 ± 0.09	0.13
Absent	27	0.39 ± 0.13	
Extrahepatic metastasis			
Present	11	0.40 ± 0.06	0.34
Absent	42	0.39 ± 0.08	
Tumor size			
> 5 cm	38	0.41 ± 0.06	0.57
≤ 5 cm	15	0.40 ± 0.09	

*classified by Edmondson-Steiner grade

**by Mann-Whitney U test, *P *< 0.05

**Table 2:** The correlation between immunohistochemical studies and clinicopathological parameters for 128 cases of HCC specimens.

Clinicopathological parameters	No. of patients	DLC2 expression		*P *value
			
		Positive	Negative	
Sex				
Male	116	30	86	0.95
Female	12	3	9	
Liver cirrhosis				
Present	80	20	60	0.79
Absent	48	13	35	
Hepatitis virus infection				
Present	107	25	82	0.15
Absent	21	8	13	
Capsule formation				
Present	71	18	53	0.91
Absent	57	15	42	
Tumor nodule				
≥ 2	82	19	63	0.36
< 2	46	14	32	
Cell differentiation*				
I ~ II	52	20	32	0.01**
III ~ IV	76	13	63	
Vein invasion				
Present	54	16	38	0.39
Absent	74	17	57	
Extrahepatic metastasis				
Present	28	9	19	0.38
Absent	100	24	76	
Tumor size				
> 5 cm	70	18	52	0.98
≤ 5 cm	58	15	43	

*classified by Edmondson-Steiner grade

**by Chi-square test, *P *< 0.05

### Correlation between DLC2 expression and RhoA expression

**Figure 2:**
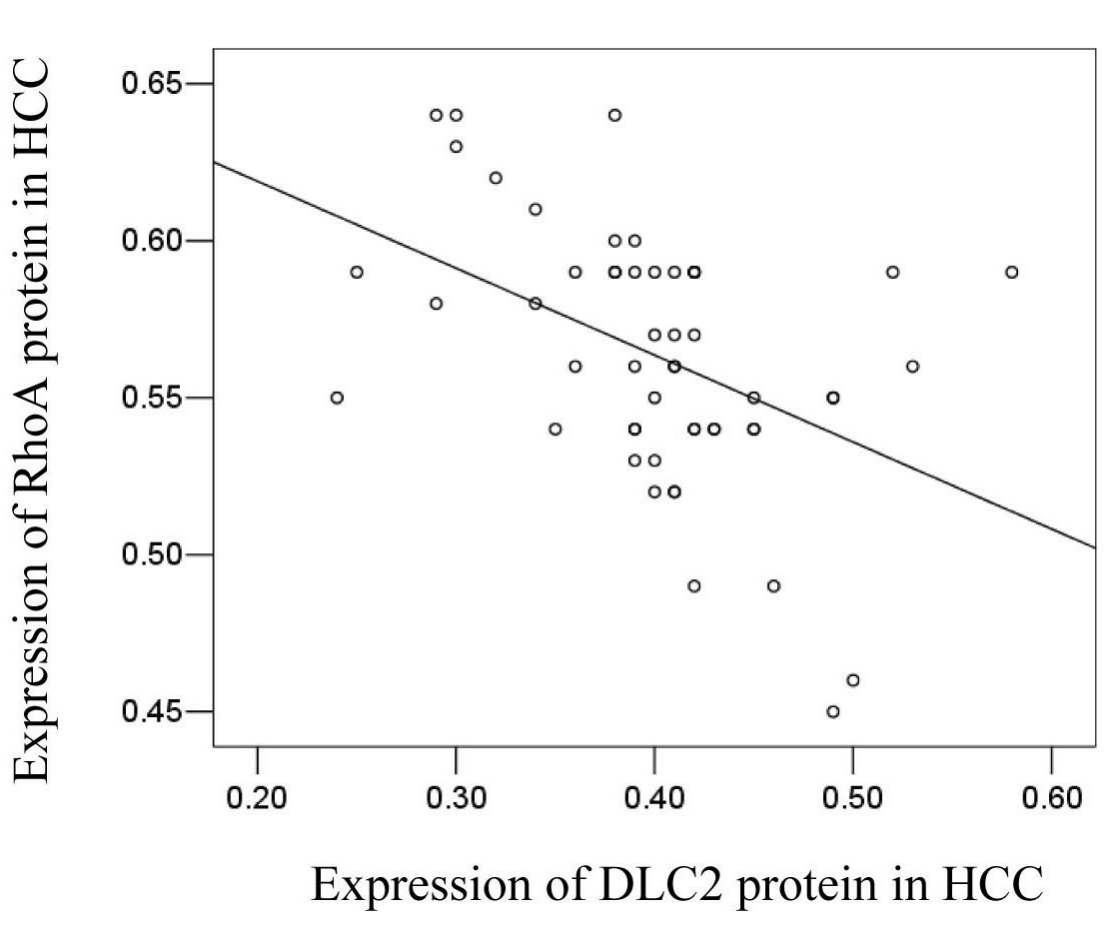
**Correlation between DLC2 protein expression and RhoA protein expression**. Correlation between DLC2 protein expression and RhoA protein expression was evaluated by Spearman's correlation coefficient. DLC2 protein levels were negative correlated with the levels of RhoA protein in HCC with adjusted -0.447 and two-tailed probability, n = 53, *P *= 0.01.

The protein expressions of DLC2 and RhoA were examined simultaneously. The protein expressions of DLC2 and RhoA showed strongly negative correlation. (correlation coefficient = -0.447, *P *= 0.01) (Figure 2).

### Correlation between DLC2 expression and prognosis of HCC

**Figure 3:**
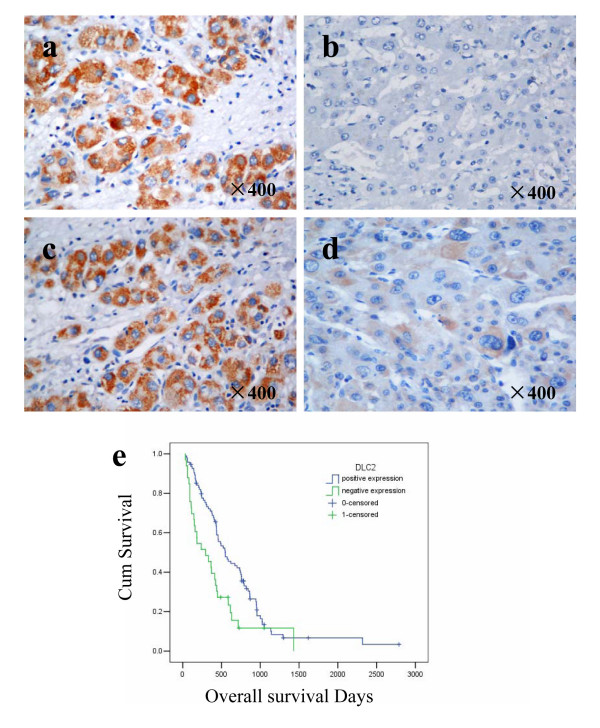
**Correlation between DLC2 expression and prognosis of HCC**. Expression of DLC2 protein was detected in 128 cases of HCC deparaffinized specimens by immunohistochemistry method. HCC specimen of DLC2 high expression confirmed by Western blotting was used as positive controls (*a*), whereas negative controls were done by omitting the primary antibody (*b*). The representative positive expression of DLC2 (*c*) and negative expression of DLC2 (*d*) in HCC specimens were presented. Kaplan-Meier survival curves for DLC2-positive expression group (n = 33) and DLC2-negative expression group (n = 95) based on results of immunohistochemistry (*e*). HCC patients with DLC2-negative expression revealed significant poor prognosis than those with DLC2-positive expression, Log-rank test, *P *= 0.003.

To further investigate the correlation between DLC2 expression level and prognosis, HCCs were divided into the DLC2 negative expression group (immunohistochemistry score 0 and 1+; n = 95) and DLC2 positive expression group (immunohistochemistry scores 2+ and 3+; n = 33). DLC2 expression level and the prognosis of HCC patients were analyzed by Kaplan-Meier method. The result showed the mean overall survival of patients with DLC2-negative expression (414 days) was significantly lower than those of patients with DLC2-positive expression (697 days, *P *= 0.003). HCC Patients with DLC2-negative expression revealed significantly poorer prognosis than those with DLC2-positive expression (Figure 3).

## Discussion

Although DLC2 has been identified as a tumor suppressor gene in HCC, the expression of DLC2 protein, especially the relationship with RhoA in clinical hepatocellular carcinoma has not been studied. The previous study demonstrated that the mRNA expression of DLC2 was significantly lower in HCC than in PCLT [13]. In human neoplasms, it has been reported that DLC2 was down-regulated in lung, ovarian, renal, breast, uterine, gastric, colon and rectal tumor [17]. Also, underexpression of DLC2 was confirmed to be help for invasion and migration of hepatocellular carcinoma cells [14]. Consistent with previous documents, our present study shows that DLC2, on protein level, is down-regulated in HCC, which indicates that DLC2 may participate in hepatocarcinogenesis.

To investigate how DLC2 affects hepatocarcinogenesis, we evaluated the correlation between DLC2 expression level and clinicopathological parameters of HCC. Our results show that the expression of DLC2 protein is lower in HCC than PCLT, which is more frequently associated with cell differentiation. This result is consistent with the role of DLC2 as a tumor suppressor gene in HCC. As previous study indicated that DLC2 encodes a RhoGAP protein with growth suppressor function in hepatocellular carcinoma, we hypothesized that DLC2 may down-regulate expression of RhoA. Thus, the protein expression of DLC2 and RhoA were examined simultaneously in human hepatocellular carcinoma. Anticipatively, the protein expression of DLC2 and RhoA showed strongly negative correlation, which was consistent with the hypothesis that DLC2 suppresses hepatocarcinogenesis by means of inhibition of RhoA activity [14]. However, there was no evidence to confirm the direct relationship between DLC2 and RhoA. The increase in RhoA protein in HCC samples observed in our study may not be a direct result of reduced DLC2 expression, but could be due to cancer-related alterations in upstream factors that regulate the two genes. Therefore, the further investigation is anticipated.

In our study, the underexpression of DLC2 correlates with cell differentiation, which suggests a possibility that DLC2 expression could be used as a potential prognostic marker for HCC patients. To explore this possibility, anti-DLC2 immunohistochemical staining was performed on 128 cases of HCC specimens. Because of lower detection sensitivity of the immunohistochemistry method, some of the 128 cases of HCC show negative staining of DLC2, notwithstanding DLC2 protein is detected in all 53 cases of HCC with western blotting. Nevertheless, when we divide the total cases of HCC into either DLC2 positive or negative group, the DLC2-positive HCC patients in general had a better prognosis than the DLC2-negative HCC patients. On the whole, our results strongly suggest that decreased DLC2 expression in HCC correlates with a poor prognosis for HCC patients.

## Conclusion

Our results strongly suggest that decreased DLC2 expression in HCC correlates with cell differentiation of HCC and overexpression of RhoA, underexpression of DLC2 in HCC indicates a poor prognosis for HCC patients.

## Competing interests

The authors declare that they have no competing interests.

## Authors' contributions

LXR conceived of the study, performed the statistical analysis and drafted the manuscript. WW carried out the immunohistochemical staining. QLY carried out the western blotting assay. YKY participated in its design and helped to draft the manuscript. All authors read and approved the final manuscript.

## Pre-publication history

The pre-publication history for this paper can be accessed here:


